# Interactions of depression, anxiety, and sleep quality with menopausal symptoms on job satisfaction among middle-aged health workers in England: a STROBE-based analysis

**DOI:** 10.1186/s12960-024-00947-4

**Published:** 2024-09-12

**Authors:** Nestor Asiamah, Camille Cronin, Joanne E. Abbott, Susan Smith

**Affiliations:** 1 Division of Interdisciplinary Research and Practice, School of Health and Social Care, University of Essex, Colchester, Essex CO4 3SQ UK; 2 Africa Centre for Epidemiology, Department of Health Services, P. O. Box AN 16284, Accra North, Ghana; 3 Division of Nursing, School of Health and Social Care, University of Essex, Colchester, Essex CO4 3SQ UK; 4 School of Health and Social Care, University of Essex, Colchester, Essex CO4 3SQ UK; 5 North-East London NHS Foundation Trust, Marsh Way Rainham, London, Greater London RM13 8GQ UK

**Keywords:** Menopausal symptoms, Job satisfaction, Depression, Anxiety, Sleep quality, Health workers, United Kingdom

## Abstract

**Background:**

This study examined the association between menopausal symptoms and job satisfaction, and ascertained whether three psychosomatic factors (e.g., anxiety, depression, and sleep quality) interact with menopausal symptoms on job satisfaction.

**Methods:**

A cross-sectional design with sensitivity analysis was adopted. The participants of the study were clinical health workers in England. Data from 154 health workers were analyzed with the hierarchical linear regression (HLR) analysis.

**Results:**

There was a negative association between menopausal symptoms and job satisfaction (*β* = −0.38; *t* = −4.81, *p* < 0.001), but this relationship became non-significant after adjusting for work stress, self-reported health, job tenure, and resilience at work. An interaction between menopausal symptoms and the psychosomatic factors was found. The strength of the negative association between menopausal symptoms and job satisfaction was weakened by sleep quality (*β* = 0.05; *t* = 0.48; *p* > 0.05) but was strengthened by anxiety (*β* = −0.22; *t* = −2.28; *p* < 0.05) and depression (*β* = −0.24; *t* = −2.16; *p* < 0.05).

**Conclusion:**

Menopausal symptoms can be directly associated with lower job satisfaction and indirectly associated with lower job satisfaction through its interaction with depression and anxiety. Menopausal symptoms can weaken the positive association between sleep quality and job satisfaction.

**Supplementary Information:**

The online version contains supplementary material available at 10.1186/s12960-024-00947-4.

## Introduction

Menopause is a stage in life when women stop having menstrual periods [1, 2] and occurs at an average age of 51 years [3]. Basic symptoms of menopause, often called menopausal symptoms, are hot flashes and night sweats [4, 5], which are common causes of anxiety, mood changes leading to irritability, skin disorders, and difficulty in sleeping [3, 6]. These experiences accompanied by menopause can adversely affect the quality of life by debilitating mental and physical health [7–9], thereby reducing the productivity of women in many spheres of life, especially at the workplace [10–12].

In the United Kingdom [UK], women comprise over 60% of the clinical workforce [13, 14]; hence, menopause can be expected to affect clinical practice in ways that reduce the quality of care. Menopause, apart from its contribution to low job satisfaction [10, 15–17], can spur turnover among health workers [12, 18]. Shore et al. [19] have reasoned that many health workers may leave their posts if they are not supported to cope with personal and work-related problems. Menopause, which is one of the problems, can incapacitate clinicians who are already working under pressure and may increase turnover [1, 20, 21], so its effective management is necessary.

Some studies [6, 22–25] have contributed to an understanding of menopausal symptoms by confirming its associations with key psychosomatic factors [i.e., sleep quality, anxiety, and depression]. Depression and anxiety are assumed to be outcomes of menopausal symptoms [4, 26], but they can also influence and exacerbate menopausal symptoms, especially if they come from multiple work stressors [e.g., low work flexibility], which is often the case among clinicians [20, 27]. Although poor sleep quality is an outcome of menopause [12, 28], interventions improving it during the menopausal transition can improve work outcomes such as job satisfaction. As such, empirical evidence regarding the interaction between menopausal symptoms and sleep quality as well as the other psychosocial factors on job satisfaction can facilitate an understanding of these potential interventions. Yet, these interactions have not been tested, and recent calls [2, 12, 18] for research on menopause imply a need for them to be assessed.

This study, therefore, tested the following four hypotheses: there is an association between menopausal symptoms and job satisfaction (hypothesis 1, H1); menopausal symptoms interact with sleep quality to influence job satisfaction (hypothesis 2, H2); menopausal symptoms interact with anxiety to influence job satisfaction (hypothesis 3, H3), and menopausal symptoms interact with depression to influence job satisfaction (hypothesis 4, H4). We tested these associations with a cross-sectional design that follows the Strengthening the Reporting of Observational Studies in Epidemiology (STROBE) checklist to guide future research and enhance the scope of the evidence. Implications for managing menopause at work to maximise job satisfaction are discussed.

Job satisfaction was chosen as an outcome variable in this study because it is one of the performance indicators mostly affected by menopause. Turnover among working women with menopause is partly due to low job satisfaction influenced by menopausal symptoms. Hence, empirical evidence on how menopausal symptoms and the psychosomatic factors influence job satisfaction among menopausal working women can inform programmes aimed at supporting this group.

## Methods

### Design

This study adopted a STROBE-based cross-sectional design including measures against Common Methods Bias (CMB).

### Nature of the study

In this study, an online survey hosted on Qualtrics was used to collect data after the participants were recruited with some inclusion criteria. Measures were taken to avoid common methods bias and confounding, and some of these measures were statistical techniques including exploratory factor analysis and hierarchical linear regression analysis.

### Study participants and their selection

The study participants were employees of a National Health Service (NHS) Trust in England. The inclusion criteria applied to select the participants were: (1) being a worker in the NHS; [2] being a woman experiencing menopause; (3) having the ability to complete the surveys in English, and (4) availability and willingness to participate in the study. The size of the eligible employee group was 6,905. We targeted women who could be experiencing different levels of menopausal symptoms and calculated the minimum sample size necessary for this study with the G*Power software and recommended statistics (i.e., effect size = 0.2; power = 0.8; significance = 0.05) [29, 30]. The minimum sample size calculated for multiple hierarchical regression with a maximum of 11 predictors was 95. Thus, a sample of 95 participants was needed to detect a minimum effect of 0.2 at 80% power for 11 predictors. Using a small effect size of 0.2 as a reference in our sample size calculation made it possible for our test to detect weak effects. We distributed the survey among all 6905 employees to maximise the response rate and the generalisability of our results. Notably, the number 6905 represents all employees of the organization, including men and women not experiencing menopause.

### Measures

We measured menopausal symptoms, anxiety, depression, and sleep quality with questionnaires or scales previously validated [12, 31]. ‘Menopausal symptoms’ was measured with the Menopause Rating Scale (MRS) adopted in whole from a previous study [31]. The MRS is associated with a five-point descriptive anchor (i.e., none – 1; mild – 2; moderate – 3; severe – 4; very severe – 5) and 11 items measuring the individual’s menopausal symptoms. Its scores range from 11 to 55, with higher scores indicating stronger menopausal symptoms. This scale produced a Cronbach’s alpha coefficient = 0.87 and was, therefore, internally consistent.

Anxiety was measured with a 7-item scale with four descriptive anchors (i.e., not difficult at all – 1, somewhat difficult – 2, very difficult – 3, and extremely difficult – 4) [12]. Its scores range from 7 to 28, where larger scores indicate higher anxiety. This scale produced a satisfactory Cronbach’s alpha coefficient = 0.91, which suggests that it was internally consistent for this study. Depression was measured with the Patient Health Questionnaire comprising 9 items and 4 descriptive anchors [i.e., not at all – 1; several days – 2; more than half the days – 3, and nearly every day – 4]. This scale produced a Cronbach’s alpha coefficient = 0.81. The scale’s scores range between 9 and 36, and higher scores represent higher symptoms of depression.

Sleep quality was measured with the Pittsburgh Sleep Quality Index [PSQI] comprising 24 items. Scores from this measure were computed based on a previous study [31]. The PSQI also produced a satisfactory internal consistency at Cronbach’s alpha coefficient = 0.76. Larger scores on the scale indicate higher sleep quality. Appendix 1 shows items used to measure anxiety, depression, sleep quality, and menopausal symptoms. We followed previously utilized methods [12] to measure job satisfaction as an indicator variable by asking the participants to rate their current level of work satisfaction in the organization based on six anchors [i.e., 0, 1, 2, 3, 4, and 5], where 0 was the lowest satisfaction level and 5 was the highest satisfaction level.

We treated and measured seven variables [e.g., age, education, general self-reported health, job tenure, work stress, resilience at work, and flexibility of work schedule] as potential confounding variables based on previous research [32–34]. Work stress was also measured by asking the participants to rate their level of stress experienced at work on the same scale as job satisfaction. Resilience at work was measured by asking the participants to indicate on a scale of 0 [lowest resilience level] to 8 [highest resilience level] their level of resilience at work. The flexibility of work schedule was measured with a categorical variable with two anchors [flexible work available – 1, and flexible work not available – 2]. Self-reported health was measured with a single item that asked the participants to rate their health status on a scale from 0 [poorest health] to 5 [best health]. Job tenure was measured by asking the participants to report the number of years they had worked in the organization. It was a categorical variable with two groups generated in our coding scheme [i.e., employees who had worked for up to 5 years – 1, and employees who had worked for more than 5 years – 2]. Education was measured by asking the participants to report their highest level of education. We recoded the levels of education reported into two groups [i.e., undergraduate education or lower – 1, and postgraduate education or higher – 2]. Following a previous study [30], we coded all categorical variables [e.g., work flexibility, job tenure, and education] into dummy-type variables.

### The questionnaire

Data were gathered with a self-reported questionnaire that contained 75 questions. The introductory part of the questionnaire presented the study’s aim and significance, ethical statements, as well as instructions for completing the survey. Another part of the questionnaire captured demographic and potential confounding variables whereas the final section presented the main variables such as menopausal symptoms. We created a digital version of the questionnaire with Qualtrics, where responses were received and later transported to the analytical software. Recommendations for limiting the response bias associated with online surveys were followed [35]. Two steps previously followed were taken to avoid or minimise CMB [30, 36]. Appendix 2 shows the steps followed and their outcomes.

### Data collection

The online survey was distributed online via Qualtrics between 19 April and 31 May 2022. A procedure used recently was followed to avoid multiple responses from the same participant [35]. The distribution of the survey was coordinated by research support personnel at the NHS Trust where the study was undertaken. A total of 167 participants completed the survey, but we discarded 13 questionnaires that were either completed by men or were completed halfway. Thus, 154 questionnaires were incorporated into the statistical analysis. The response rate of this study was 2%, but this proportion is underestimated since the number of employees in the organization (i.e., 6,905) included men and women not experiencing menopause.

### Statistical analyses

The SPSS version 28 was used to analyze the data in two major stages, an exploratory phase and the hypotheses testing phase. In the exploratory phase, we summarised the data with descriptive statistics, and assessed assumptions relevant to hierarchical linear regression [HLR] analysis, which was used to test the hypotheses and perform the first sensitivity analyses. The missing data were not removed since they were less than 3% of the data and were randomly distributed across the variables [30, 37].

As part of the exploratory analysis, we ascertained whether our data met five assumptions governing HLR. Appendix 3 shows these assumptions and the specific steps taken to assess and meet them. The final aspect of the exploratory analysis was the first sensitivity analysis performed to screen all measured potential confounders for the ultimate confounding variables. In harmony with previous studies [30, 37], this analysis aimed to identify only the variables likely to confound the hypothesised relationships. Self-reported health, job tenure, stress, and resilience were identified in this analysis as ultimate confounders. Appendix 4 shows the specific steps taken in this sensitivity analysis following previous research [30].

The second phase of the analysis was focused on testing the study's four hypotheses. We fitted two groups of models through HLR analysis. The first group comprised the baseline or unadjusted regression models, which did not include the ultimate confounders retained in the first sensitivity analysis. This group has four models, with the first model assessing the association between menopausal symptoms and job satisfaction whereas models 2, 3 and 4 tested the associations between three interaction terms [i.e., MRSxAnxiety, MRSxDepression, and MRSxSleepQual] and job satisfaction. These interaction terms were created following a recent study [37] with the ‘compute variable’ function.

The second group of models comprised four ultimate or adjusted models on which this study’s conclusions were based. These models built upon the unadjusted models by incorporating the ultimate confounders and, therefore, present associations not biased by confounders. Following previous studies [30, 37], a second sensitivity analysis was performed by comparing the corresponding unadjusted models [i.e., models 1–4] and adjusted models [i.e., models 5–8] to understand the influence of the ultimate confounders on the adjusted models. The statistical significance of the results was detected at a minimum of p < 0.05.

Interactions were assessed to understand how much: [1] the psychosomatic factors changed the strength of the association between menopausal symptoms and job satisfaction, and [2] menopausal symptoms changed the strength of the associations between the psychosomatic factors and job satisfaction. Appendix 5 shows the steps taken to assess the interactions based on complementary results in Appendix 6.

## Results

Table 1 shows summary statistics on all variables included in the study. In this table, 29% [n = 44] of the participants had worked for up to 5 years whereas 71% [*n* = 110] had worked for more than 5 years. The average age of the participants was about 47 years. The average ‘menopausal symptoms’ was about 25 [Mean = 25.14; SD = 8.68].

**Table 1:** Summary statistics on variables measured

Variable	Group	n/Mean	%/SD
Categorical demographic variables			
Education	Undergraduate education or lower	62	40%
	Postgraduate education or higher	92	60%
	Total	154	100%
Job tenure	≤ 5 yrs	44	29%
	> 5 yrs	110	71%
	Total	154	100%
Flexible work available	Available	132	86%
	Not available	22	14%
	Total	154	100%
Continuous demographic variables			
Age (yrs)	–	47.3	9.75
Self-reported health	–	3.66	0.97
Stress	–-	3.02	1.21
Resilience	–-	5.64	1.66
Anxiety	–-	13.99	5.44
Depression	–-	16.51	5.55
Sleep quality	–-	24.50	3.25
Menopausal symptoms	–-	25.14	8.68
Job satisfaction	–-	3.76	1.07

–Not applicable; n – frequency; SD – standard deviation; Min – minimum value from the scale; Max – maximum value from the scale

Table 2 shows a moderate negative correlation between menopausal symptoms and job satisfaction [r = −0.378; *p* < 0.001; two tailed], which suggests that higher menopausal symptoms were associated with lower job satisfaction. There was a strong positive association between menopausal symptoms and anxiety [*r* = 0.657; *p* < 0.001; two tailed] as well as depression [*r* = 0.729; *p* < 0.001; two tailed], which means that higher depression symptoms and anxiety were associated with higher menopausal symptoms.

**Table 2:** The bivariate correlations between menopausal symptoms, depression, anxiety, sleep quality, and the ultimate confounders

Variable	1	2	3	4	5	6	7	8	9
1. MRS	1	−0.378**	0.657**	0.729**	−0.186*	−0.500**	0.167*	0.429**	−0.282**
2. Job satisfaction		1	−0.309**	−0.350**	0.519**	0.313**	−0.11	−0.297**	0.208*
3. Anxiety			1	0.718**	−0.139	−0.370**	0.066	0.275**	−0.242**
4. Depression				1	−0.319**	−0.521**	0.082	0.311**	−0.368**
5. Sleep quality					1	0.275**	−0.086	0.169*	0.698**
6. General health						1	−0.153	−0.198*	0.270**
7. Job tenure***							1	0.078	−0.161*
8. Stress								1	−0.006
9. Resilience									1

**p < 0.001; *p < 0.05; MRS – menopausal symptoms; ***The group ‘≤ 5 years’ was set as reference to job tenure

In model 1 of Table 3, a negative relationship between menopausal symptoms and job satisfaction [*β* = −0.38; *t* = −4.81; *p* < 0.001] was confirmed. The three interaction terms were also negatively associated with job satisfaction at *p* < 0.001 [See Models 2, 3 and 4]. After adjusting for the ultimate confounders, the association between menopausal symptoms and job satisfaction became non-significant at *p* > 0.05 [*β* = −0.18; *t* = −1.83] [see Model 5]. We refer to this association subsequently as the *primary association* and its coefficient [i.e., *β* = −0.18] as the *primary coefficient*. The standardized coefficients of ‘MRSxAnxiety’ and ‘MRSxDepression’ on job satisfaction in models 6 and 7, respectively, are significant at *p* < 0.05, but the standardized coefficient of ‘MRSxSleepQual’ on job satisfaction is non-significant in model 8. In model 6, the coefficient associated with the interaction term ‘MRSxAnxiety’ is −0.22, which is 22% or 0.04 [in absolute terms] higher than the primary coefficient [i.e., *β* = −0.18] in model 5. The coefficient corresponding to ‘MRSxDepression’ [*β* = −0.24] is 33% or 0.06 [in absolute terms] higher than the primary coefficient whereas the coefficient of ‘MRSxSleepQual’ [*β* = 0.05] is 0.13 [72%] lower than the primary coefficient. The above interactions suggest that the association between menopausal symptoms and job satisfaction is strengthened by 22% and 33% by anxiety and depression respectively whereas it is weakened by 72% by sleep quality.

**Table 3:** The interactions of anxiety, depression, and sleep quality with menopausal symptoms on job satisfaction

Model	Predictor	Coefficients				Model fit			
		B	SE	Beta(*t*)	95% CI	R^2^	Adjusted R^2^	Durbin Watson	F statistic
Baseline (unadjusted models)									
1	(Constant)	4.890	0.254	(19.27)**	± 1.00	0.143	0.137	–	23.15**
	Menopausal symptoms	−0.046	0.010	−0.38(−4.81)**					
2	(Constant)	4.374	0.148	(29.63)**	± 0.58	0.165	0.159	–	26.82**
	MRSxAnxiety	−0.002	0.000	−0.41(−5.18)**	± 0.00				
3	(Constant)	4.379	0.156	(28.07)**	± 0.62	0.162	0.156	–	25.67**
	MRSxDepression	−0.001	0.000	−0.40(−5.07)**	± 0.00				
4	(Constant)	4.273	0.266	(16.09)**	± 1.05	0.032	0.025	–	4.59*
	MRSxSleepQual	−0.001	0.000	−0.18(−2.14)*	± 0.00				
Ultimate (adjusted) models									
5	(Constant)	3.921	0.712	(5.51)**	± 2.82	0.198	0.167	1.87	6.42**
	Menopausal symptoms	−0.023	0.012	−0.18(−1.83)	± 0.05				
	General health	0.154	0.102	0.14(1.51)	± 0.40				
	Job tenure***	−0.041	0.067	−0.05(−0.62)	± 0.27				
	Stress	−0.159	0.078	−0.18(−2.04)*	± 0.31				
	Resilience	0.082	0.055	0.12(1.48)	± 0.22				
6	(Constant)	3.835	0.650	(5.90)**	± 2.57	0.223	0.192	1.86	7.29**
	MRSxAnxiety	−0.001	0.000	−0.22(−2.28)*	± 0.00				
	General health	0.155	0.104	0.14(1.49)	± 0.41				
	Job tenure***	−0.038	0.067	−0.05(–0.56)	± 0.27				
	Stress	−0.175	0.077	−0.19(−2.28)*	± 0.30				
	Resilience	0.067	0.056	0.10(1.20)	± 0.22				
7	(Constant)	3.946	0.726	(5.44)**	± 2.87	0.196	0.163	2.02	6.04**
	MRSxDepression	−0.001	0.000	−0.24(−2.16)*	± 0.00				
	General health	0.112	0.117	0.10(0.95)	± 0.46				
	Job tenure***	−0.043	0.069	−0.05(−0.63)	± 0.27				
	Stress	−0.141	0.080	−0.16(−1.75)	± 0.32				
	Resilience	0.064	0.058	0.10(1.10)	± 0.23				
8	(Constant)	3.047	0.638	(4.78)**	± 2.52	0.179	0.147	1.87	5.67**
	MRSxSleepQual	0.000	0.000	0.05(0.48)	± 0.00				
	General health	0.247	0.099	0.22(2.49)*	± 0.39				
	Job tenure***	−0.053	0.068	−0.06(−0.78)	± 0.27				
	Stress	−0.231	0.080	−0.26(−2.88)*	± 0.32				
	Resilience	0.099	0.055	0.15(1.79)	± 0.22				

**p < 0.001; *p < 0.05; ***The group ‘ ≤ 5 years’ was set as reference to job tenure; SE – standard error (of B); CI – confidence interval; each predictor produced a variance inflation factor < 3 and a tolerance value ≥ 0.6

Models 4, 5, and 6 in Appendix 6 show the adjusted models evaluating the influences of the psychosomatic factors on job satisfaction. Models 1–3 of this appendix show the corresponding non-adjusted models. The coefficient of ‘MRSxAnxiety’ in model 6 of Table 3 is 36% higher than the coefficient between anxiety and job satisfaction [i.e., *β* = −0.14] in model 4 of Appendix 6. Thus, the association between anxiety and job satisfaction is strengthened by 36% by menopausal symptoms. The coefficient of ‘MRSxDepression’ in model 7 of Table 3 is 29% higher than the coefficient between depression and job satisfaction [i.e., *β* = −0.17] in model 5 of Appendix 6. This result suggests that the association between depression and job satisfaction is strengthened by 29% by menopausal symptoms. Finally, the coefficient of ‘MRSxSleepQual’ in model 8 of Table 3 is 94% lower than the coefficient between sleep quality and job satisfaction [i.e., *β* = 0.899] in model 6 of Appendix 6. This result suggests that the association between sleep quality and job satisfaction was weakened by 94% and made non-significant by menopausal symptoms.

**Fig. 1:**
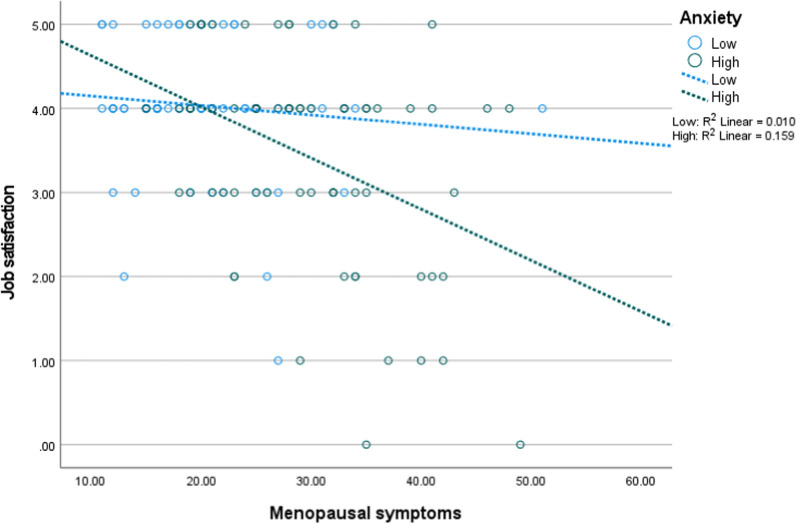
Interaction of anxiety with menopausal symptoms on job satisfaction (n = 154, low = 77, high = 77)

**Fig. 2:**
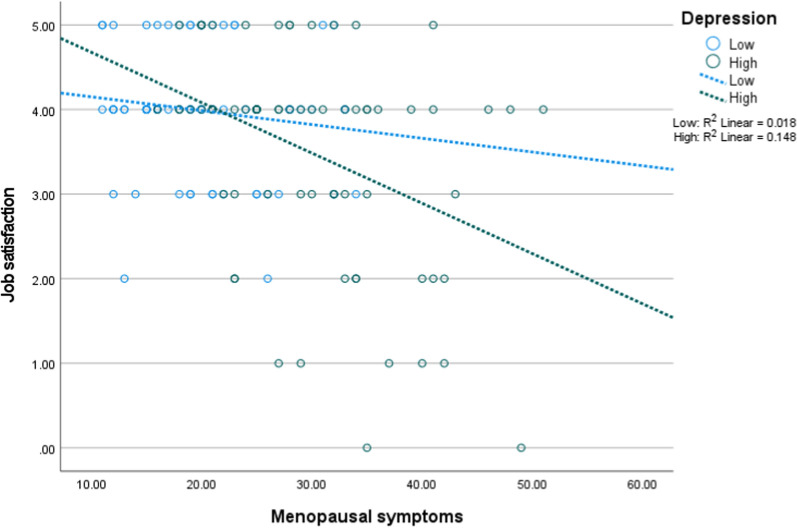
Interaction of depression with menopausal symptoms on job satisfaction (n = 154; low = 77, high = 77)

**Fig. 3:**
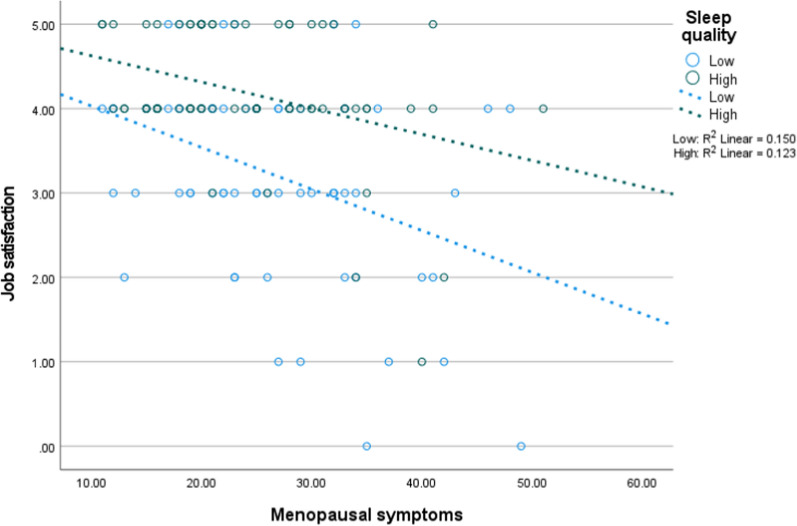
Interaction of sleep quality with menopausal symptoms on job satisfaction (low = 77, high = 77, n = 154)

Figures 1, 2 and 3 depict the interaction of menopausal symptoms with the psychosomatic factors on job satisfaction. In Fig. 1, higher menopausal symptoms were associated with low job satisfaction at different levels of anxiety. In Fig. 2, higher menopausal symptoms were associated with low job satisfaction at different levels of sleep quality. In Fig. 3, higher menopausal symptoms were associated with low job satisfaction at different levels of sleep quality, but this interaction became non-significant after adjusting for the ultimate covariates.

## Discussion

This study assessed the association between menopausal symptoms and job satisfaction as well as the interactions between three psychosomatic factors and menopausal symptoms on job satisfaction in England.

This study found no association between menopausal symptoms and job satisfaction after adjusting for the ultimate confounders, which means the first hypothesis [H1] was not confirmed. This result implies that some other work-related factors including stress and general self-reported health may influence lower job satisfaction through menopausal symptoms. ‘Menopausal symptoms’ is more likely to influence job satisfaction in a situation where clinicians are already experiencing poor health [possibly from other comorbidities or job factors] and a high level of work-related stress. A body of studies [27, 38, 39] has reported an increase in work-related stress and a reduction in health among clinicians during the COVID-19 pandemic. It is, therefore, possible that the above result was influenced by clinicians’ increased workload and COVID-19-related circumstances. In contrast to our result, research suggests that menopausal symptoms can be associated with a low level of job satisfaction among clinical workers [11, 12, 17, 40]. In some studies that used a qualitative design [11, 12, 41], health workers shared lived experiences that imply a negative association between menopausal symptoms and job satisfaction, but these studies did not examine the association between menopausal symptoms and job satisfaction nor adjusted for the ultimate confounders.

This study found an interaction between menopausal symptoms and sleep quality on job satisfaction, thereby confirming the second hypothesis [H2]. This result suggests that sleep quality is less likely to enhance job satisfaction at higher menopausal symptoms. It is rooted in the negative association between menopausal symptoms and sleep quality, which has been confirmed by several studies [6, 25, 28]. In China, for example, Zhang and colleagues found a negative association between menopausal symptoms and sleep quality among a sample of women with menopausal symptoms. This result was confirmed in Korea [25] and Spain, though the result reported in the context of Spain by Zagalaz-Anula et al. was focused on post-menopausal women. One of the unique attributions of this study is our extension of this confirmed relationship to job satisfaction, enabling us to advance the ongoing debate and identify implications reported later in this paper.

An interaction between anxiety and menopausal symptoms on job satisfaction was found, which supports the third hypothesis [H3]. This result suggests that the potential negative influence of menopausal symptoms on job satisfaction is stronger at higher anxiety. This nexus is also rooted in the negative association between menopausal symptoms and anxiety, which has been confirmed in the extant literature [24, 26, 42]. In Australia, Mulhall et al. [26] confirmed a positive association between menopausal symptoms and anxiety. This evidence has been affirmed in Taiwan with a cross-sectional study [25] and with a systematic review carried out by Bryant et al [42]. An interaction between menopausal symptoms and depression on job satisfaction was also confirmed in this study, which means the fourth hypothesis [H4] is supported by our data. This result implies that the potential negative influence of depression on job satisfaction is stronger at higher menopausal symptoms, or the potential negative influence of menopausal symptoms on job satisfaction is stronger at higher depressive symptoms. This interaction, like the two confirmed earlier, stems from a positive association between menopausal symptoms and depression, which has been consistently confirmed in research [23, 24, 43]. Suffice it to say that interactions of depression and anxiety with menopausal symptoms can be associated with lower job satisfaction, which has important implications for theory and practice.

Quite pronounced in the literature is the implication of our result for the Job Demands-Resources [JD-R] theory [21, 44], which posits that burnout, stress, and their work outcomes [e.g., dissatisfaction, low job satisfaction] are likely to occur if job demands are more than job resources. Job demands in this context include any physical, social, organizational, or psychological aspects of the job that limit the employee’s ability to accomplish job tasks [21]. Work pressure, anxiety, depression, and other related conditions [i.e., menopause] are part of these demands [21, 44]. Our findings suggest that anxiety and depression are demands that can interact with a third demand, menopausal symptoms. Since this interaction affects work-related variables such as job satisfaction, organizations and employers are expected to recognise menopausal symptoms and any variable that interacts with it to affect job outcomes as job demands. Sleep quality, on the other hand, can be a resource for desired work outcomes such as satisfaction and productivity [45, 46] if interventions are put in place by the organization to enhance it during the menopausal transition. These interventions are necessary because poor sleep quality is one of the most frequently reported experiences of menopausal women [3, 4, 7].

The interaction between menopausal symptoms and two of the psychosomatic factors [i.e., anxiety and depression] on job satisfaction suggests that menopausal symptoms may more strongly reduce job satisfaction due to higher levels of anxiety and depression. Higher levels of these two psychosomatic conditions can also be supported by menopausal symptoms to maximise a fall in job satisfaction. This being so, low job satisfaction and its possible outcomes [e.g., turnover, work absenteeism, underperformance] are more likely to occur among clinicians who are experiencing multiple psychosomatic conditions such as anxiety and depression. The literature to date suggests that health workers usually experience anxiety, depression, and stress concurrently on the job [19, 20, 27], which means that the foregoing interactions may result in job dissatisfaction or a low job satisfaction.

Among the ultimate confounders, general health and work stress have the strongest correlations with menopausal symptoms [see Table 2], which implies that these two factors alone could maximise the influence of menopausal symptoms on job satisfaction. There may be a need for organizations, as part of their menopause management programmes, to roll out interventions to optimise general staff health and enable individuals to avoid or cope with work stress. As the correlation between resilience at work and menopausal symptoms also suggests [see Table 2], organizations should be cognisant of individual-level capabilities such as resilience that could enable clinicians to maintain job satisfaction despite their experience of menopause. This viewpoint is corroborated by studies [47, 48] that have acknowledged a need for clinical staff to be equipped with resilience.

Our results also imply the necessity of other menopause management programmes that provide educational resources (i.e., a working group of peers who share information about menopause), counselling, and health services in the organization. Resilience training and initiatives providing mental health support for menopausal women may reduce anxiety, depression, and menopausal symptoms. This effort may reduce the negative impact of menopausal symptoms on job satisfaction. Organizational efforts aimed at improving sleep quality among women with menopause are imperative. These efforts may include fostering work-life balance and creating a supportive work environment where menopausal women are supported. Finally, we recommend continuous monitoring of the health of menopausal women and regular assessment of their menopausal symptoms and job satisfaction to develop interventions meeting their changing needs.

We acknowledge that our study has some limitations. Though a robust method was employed to adjust for confounding variables, our cross-sectional design could not have established causation between the variables. Hence, future researchers are encouraged to employ longitudinal or experimental designs and control for other factors likely to confound the relationship tested. These factors can be identified in a systematic review based on the study context. Our sample size was relatively small due to the study’s low response rate, although the minimum sample necessary for this study was obtained and used. As such, the findings of this study could have limited generalisability. The small sample used in this study poses other limitations, including increased Type I and Type II errors as well as hindered ability to perform subgroup analyses. Although based on a previous study undertaken in the UK [12], our use of single-item measures for some of the variables possibly did not capture relevant domains of these variables. We, therefore, encourage future researchers to use Likert scales to measure such variables as constructs. Self-reported measures vulnerable to different forms of bias (e.g., recall, and social desirability) were utilised in this study. As such, future researchers may use objective measures if possible. Despite these limitations, this study has several strengths.

This study was the first to evaluate the interactions between menopausal symptoms and the three psychosomatic factors, thereby improving an understanding of potential indirect influences of menopausal symptoms on job satisfaction. Our robust statistical technique against confounding and measures against CMB enabled us to minimise or avoid common threats to internal validity. Finally, we adopted a STROBE-based design, which means that this study meets all recommendations for designing and reporting a cross-sectional design. Appendix 7 shows the STROBE checklist. We chose a HLR analysis over a traditional multiple linear regression analysis because it enabled us to perform our sensitivity analysis to screen for the ultimate confounders. With this approach, we were able to show any changes in the effect sizes due to the ultimate confounders and avoid incorporating irrelevant potential confounders into our analysis. We could not have performed this stepwise analysis with a traditional multiple linear regression analysis.

## Conclusion

The study concludes that ‘menopausal symptoms’ may not only be directly associated with lower job satisfaction but can also be indirectly associated with lower job satisfaction through its interaction with depression and anxiety. Finally, menopausal symptoms can weaken the positive association between desired conditions such as high sleep quality and job satisfaction. This study was the first to assess the interaction of menopausal symptoms and the psychosomatic factors on job satisfaction. By treating job satisfaction as an outcome variable, this study provides evidence that can inform programmes for supporting working women experiencing menopause.

## Supplementary Information

Additional file 1.

Additional file 2.

Additional file 3.

Additional file 4.

Additional file 5.

Additional file 6.

Additional file 7.

## Data Availability

The data used for this manuscript are attached as supplementary material. The file name is "Data".

## Abbreviations

CMB: Common Methods Bias
HLR: Hierarchical Linear Regression
MRS: Menopause Rating Scale
PSQI: Pittsburgh Sleep Quality Index
SD: Standard deviation
STROBE: Strengthening the Reporting of Observational Studies in Epidemiology
UK: United Kingdom
