# Supplementary material for: Interactions of depression, anxiety, and sleep quality with menopausal symptoms on job satisfaction among middle-aged health workers in England: a STROBE-based analysis
Source: Hum Resour Health. 2024 Sep 12;22:64. doi: 10.1186/s12960-024-00947-4 (PMC11396863; doi:10.1186/s12960-024-00947-4)
Supplement: Supplementary file 1 — Additional file 1. [file 12960_2024_947_MOESM1_ESM.doc]

APPENDIX 1

Appendix 1a. Items for measuring sleep quality

| Number | Item |
| --- | --- |
| 1 | During the past month, what time have you usually gone to bed? - Please use 24 hour format e.g. 2200 |
| 2 | During the past month, how long (in minutes) has it usually taken you to fall asleep each night? - Please state minutes e.g. 30 |
| 3 | During the past month, what time have you usually gotten up in the morning? - Please use 24 hour format e.g. 0700 |
| 4 | Hours slept |
| 5 | During the past month, how often have you had trouble sleeping because you... - Cannot get to sleep within 30 minutes |
| 6 | During the past month, how often have you had trouble sleeping because you... - Wake in the middle of the night or early morning |
| 7 | During the past month, how often have you had trouble sleeping because you... - Have to get up to use the bathroom |
| 8 | During the past month, how often have you had trouble sleeping because you... - Cannot breathe comfortably |
| 9 | During the past month, how often have you had trouble sleeping because you... - Cough or snore loudly |
| 10 | During the past month, how often have you had trouble sleeping because you... - Feel too cold |
| 11 | During the past month, how often have you had trouble sleeping because you... - Feel too hot |
| 12 | During the past month, how often have you had trouble sleeping because you... - Have bad dreams |
| 13 | During the past month, how often have you had trouble sleeping because you... - Have pain |
| 14 | Use of sleep medication |
| 15 | During the past month... - How often have you had trouble staying awake while driving, eating meals, or engaging in social activity? |
| 16 | During the past month... - How much of a problem has it been for you to keep up enough enthusiasm to get things done? |
| 17 | During the past month... - How much quality sleep did you have? |
| 18 | Do you have a bed partner or room mate? |
| 19 | If you do have a room mate or bed partner, please ask them how often in the past month you have had: - Loud snoring |
| 20 | If you do have a room mate or bed partner, please ask them how often in the past month you have had: - Long pauses between breaths while asleep |
| 21 | If you do have a room mate or bed partner, please ask them how often in the past month you have had: - Legs twitching or jerking while you sleep |
| 22 | If you do have a room mate or bed partner, please ask them how often in the past month you have had: - Episodes of disorientation or confusion during sleep |
| 23 | If you do have a room mate or bed partner, please ask them how often in the past month you have had: - Other restlessness while you sleep, please describe: |
| 24 | If you do have a room mate or bed partner, please ask them how often in the past month you have had: - Other restlessness while you sleep, please describe: - Text |

**Appendix 1b. Items for measuring menopausal symptoms**

Which of the following symptoms apply to you at this time? Please mark the appropriate box for each symptom. For symptoms that do not apply, please mark 'none'.

| Number | Item |
| --- | --- |
| 1 | Hot flushes, sweating (episodes of sweating) |
| 2 | Heart discomfort (unusual awareness of heart beat, heart skipping, heart racing, tightnes |
| 3 | Sleep problems (difficulty falling asleep, difficulty in sleeping through, waking up earl |
| 4 | Depressive mood (feeling down, sad, on the verge of tears, lack of drive, mood swings) |
| 5 | Irritability (feeling nervous, inner tension, feeling aggressive) |
| 6 | Anxiety (inner restlessness, feeling panicky) |
| 7 | Physical and mental exhaustion (general decrease in performance, impaired memory, decreas |
| 8 | Bladder problems (difficulty in urinating, increased need to urinate, bladder incontinenc |
| 9 | Sexual problems (change in sexual desire, in sexual activity and satisfaction) |
| 10 | Dryness of vagina (sensation of dryness or burning in the vagina, difficulty with sexual |
| 11 | Joint and muscular discomfort (pain in the joints, rheumatoid complaints) |

**Appendix 1c. Items for measuring depression**

Over the last two weeks, how often have you been bothered by the following problems? …

| Number | Item |
| --- | --- |
| 1 | Feeling nervous, anxious or on edge |
| 2 | Not being able to stop or control worrying |
| 3 | Worrying too much about different things |
| 4 | Trouble relaxing |
| 5 | Been so restless that it is hard to sit still |
| 6 | Becoming easily annoyed or irritable |
| 7 | Feeling afraid, as if something awful might happen |

**Appendix 1d. Items for measuring anxiety**

Over the last two weeks, how often have you been bothered by the following problems?

| Number | Item |
| --- | --- |
| 1 | Little interest or pleasure in doing things |
| 2 | Feeling down, depressed or hopeless |
| 3 | Trouble falling/staying asleep sleeping too much |
| 4 | Feeling tired or having little energy |
| 5 | Over the last 2 weeks, how often have you been bothered by any of the following problems? - 5. Poor appetite or overeating |
| 6 | Feeling bad about yourself or that you are a failure or have let yourself or your family down |
| 7 | Trouble concentrating on things, such as reading the newspaper or watching television. |
| 8 | Moving or speaking so slowly that other people could have noticed. Or the opposite; being so fidgety or restless that you have been moving around a lot more tha |
| 9 | Thoughts that you would be better off dead or of hurting yourself in some way. |
