# Supplementary material for: Interactions of depression, anxiety, and sleep quality with menopausal symptoms on job satisfaction among middle-aged health workers in England: a STROBE-based analysis
Source: Hum Resour Health. 2024 Sep 12;22:64. doi: 10.1186/s12960-024-00947-4 (PMC11396863; doi:10.1186/s12960-024-00947-4)
Supplement: Supplementary file 2 — Additional file 2. [file 12960_2024_947_MOESM2_ESM.doc]

Appendix 2. Steps followed to assess common methods bias

| # | Steps |
| --- | --- |
| Steps at the stage of study design | |
| 1 | Questionnaire was structured to make each section distinct |
| 2 | Instructions for completing each section of the questionnaire were provided in the questionnaire |
| 3 | Research assistants and researchers provided clarifications to the participants when required |
| Statistical technique | |
| 4 | Exploratory factor analysis (with varimax rotation) was used to explore the factor structure of the scales following the one-factor method |
| 5 | Common methods bias is absent if each scale produced more than a factor. |
| 6 | The EFA produced factor solutions with at least two factors as follows: anxiety (2 factors extracted, total variance = 60.0%, factor loading ≥0.5); depression (2 factors extracted; total variance = 64.4%; factor loading ≥0.5); sleep quality (3 factors extracted; total variance = 56.5%; factor loading ≥0.5), and menopausal symptoms (2 factors extracted; total variance = 57.3%; factor loading ≥0.5). |

**Note:** The above steps were adopted from Jakobsen and Jensen (2015)
