# Supplementary material for: Interactions of depression, anxiety, and sleep quality with menopausal symptoms on job satisfaction among middle-aged health workers in England: a STROBE-based analysis
Source: Hum Resour Health. 2024 Sep 12;22:64. doi: 10.1186/s12960-024-00947-4 (PMC11396863; doi:10.1186/s12960-024-00947-4)
Supplement: Supplementary file 3 — Additional file 3. [file 12960_2024_947_MOESM3_ESM.doc]

Appendix 3. Steps taken to assess and meet assumptions for performing a hierarchical linear regression analysis

| Assumption | Step | Result | Decision |
| --- | --- | --- | --- |
| Normality of the data associated with the dependent variable | We estimated kurtosis and skewness and performed the Shapiro-Wilk’s test on the dependent variable (job satisfaction). | The Shapiro-Wilk’s test was non-significant at p>0.05. The kurtosis and skewness statistics were also within the recommended ranges (Garson, 2012). | Normality of the data was confirmed |
| Linearity of the hypothesized relationships | We plotted standardized residuals against standardized predicted values of the dependent variable in the first ultimate model or model 5. | A pattern of the graph recommended by Garson (2012) was observed. | Linearity was confirmed |
| Independence of errors | We computed the Durbin-Watson statistic through all multiple regression models | Durbin-Watson statistic was approximately 2 as recommended (Garson, 2012) | Independence of errors was met |
| Multi-collinearity | Tolerance values were computed for all predictors through the multiple regression model | The tolerance values produced were >0.2 as recommended (Bempong and Asiamah, 2022) | Multicollinearity was absent |
| Homoscedasticity | We plotted standardized residuals against standardized predicted values of the dependent variable in model 5 or the first ultimate model | The graph produced a satisfactory pattern recommended by Garson (2012) | The assumption was met |
