# Supplementary material for: Interactions of depression, anxiety, and sleep quality with menopausal symptoms on job satisfaction among middle-aged health workers in England: a STROBE-based analysis
Source: Hum Resour Health. 2024 Sep 12;22:64. doi: 10.1186/s12960-024-00947-4 (PMC11396863; doi:10.1186/s12960-024-00947-4)
Supplement: Supplementary file 4 — Additional file 4. [file 12960_2024_947_MOESM4_ESM.doc]

Appendix 4. The specific steps taken to perform the sensitivity analysis for the ultimate confounding variables

| Stage | Step | Assumption |
| --- | --- | --- |
| 1 | 1 | We fitted a simple linear regression model to assess the relationship between menopausal symptoms and job satisfaction |
|  | 2 | The standardised regression weight from step 1 was noted |
|  | 3 | We fitted a multiple linear regression model in which all measured confounding variables were treated as predictors of the main independent variable, menopausal symptoms |
|  | 4 | We identified from step 3 potential confounders that have a p-value ≥0.25 |
|  | 5 | We removed predictors that produced a p≥0.25 at step 4, and the other predictors were kept for the next stage of the analysis |
| 2 | 6 | We adjusted for each of the remaining confounding variables in the model fitted at step 1 |
|  | 7 | We compute the per cent change between the standardised regression weight at step 1 and the new weight resulting from step 6 |
|  | 8 | All potential confounders that produce a change of 10% or more were incorporated into the final analysis as the ultimate confounders |
