# Supplementary material for: Interactions of depression, anxiety, and sleep quality with menopausal symptoms on job satisfaction among middle-aged health workers in England: a STROBE-based analysis
Source: Hum Resour Health. 2024 Sep 12;22:64. doi: 10.1186/s12960-024-00947-4 (PMC11396863; doi:10.1186/s12960-024-00947-4)
Supplement: Supplementary file 5 — Additional file 5. [file 12960_2024_947_MOESM5_ESM.doc]

Appendix 5. Steps taken to assess the interactions

1. We compared the standardised regression weight between menopausal symptoms and job satisfaction in model 5 to the weights of the interaction terms in models 6, 7 and 8.
2. We tested the associations between menopausal symptoms and the psychosomatic factors (i.e., anxiety, depression, and sleep quality) through adjusted and unadjusted models (see Appendix 6).
3. We then compared the standardised weight corresponding to anxiety (see model 4, Appendix 6) to the standardised weight of the interaction between anxiety and menopausal symptoms (see model 6, Table 3).
4. The standardised weight corresponding to depression (see model 5, Appendix 6) is compared to the standardised weight of the interaction between depression and menopausal symptoms (see model 7, Table 3).
5. The standardised weight corresponding to sleep quality (see model 6, Appendix 6) is compared to the standardised weight of the interaction between sleep quality and menopausal symptoms (in model 8, Table 3).
