# Supplementary material for: Interactions of depression, anxiety, and sleep quality with menopausal symptoms on job satisfaction among middle-aged health workers in England: a STROBE-based analysis
Source: Hum Resour Health. 2024 Sep 12;22:64. doi: 10.1186/s12960-024-00947-4 (PMC11396863; doi:10.1186/s12960-024-00947-4)
Supplement: Supplementary file 6 — Additional file 6. [file 12960_2024_947_MOESM6_ESM.doc]

Appendix 6. The influences of anxiety, depression, and sleep quality on job satisfaction

| Model | Predictor | Coefficients | | | | | 95% CI |
| --- | --- | --- | --- | --- | --- | --- | --- |
|  |  | B | SE | Beta | t |  |  |
| Non-adjusted models | | | | | | | |
| 1 | (Constant) | 4.613 | 0.237 | --- | 19.443** | <.001 | ±0.938 |
|  | Anxiety | -0.06 | 0.016 | -0.309 | -3.836** | <.001 | ±0.062 |
| 2 | (Constant) | 4.87 | 0.274 | --- | 17.803** | <.001 | ±1.082 |
|  | Depression | -0.069 | 0.016 | -0.35 | -4.358** | <.001 | ±0.062 |
| 3 | (Constant) | -0.965 | 0.658 | --- | -1.467 | 0.145 | ±2.600 |
|  | Sleep quality | 0.19 | 0.026 | 0.519 | 7.228** | <.001 | ±0.104 |
| Adjusted models | | | | | | | |
| 4 | (Constant) | 3.667 | 0.682 | --- | 5.374** | <.001 | ±2.699 |
|  | Anxiety | -0.027 | 0.017 | -0.137 | -1.558 | 0.122 | ±0.068 |
|  | General health | 0.226 | 0.098 | 0.201 | 2.304* | 0.023 | ±0.389 |
|  | Job tenure | -0.04 | 0.067 | -0.047 | -0.593 | 0.554 | ±0.265 |
|  | Stress | -0.204 | 0.074 | -0.225 | -2.75* | 0.007 | ±0.293 |
|  | Resilience | 0.076 | 0.056 | 0.113 | 1.363 | 0.175 | ±0.22 |
| 5 | (Constant) | 3.959 | 0.831 | --- | 4.764** | <.001 | ±3.288 |
|  | Depression | -0.033 | 0.02 | -0.167 | -1.635 | 0.105 | ±0.08 |
|  | General health | 0.173 | 0.113 | 0.149 | 1.536 | 0.127 | ±0.446 |
|  | Job tenure | -0.05 | 0.069 | -0.059 | -0.729 | 0.467 | ±0.272 |
|  | Stress | -0.166 | 0.077 | -0.183 | -2.145* | 0.034 | ±0.306 |
|  | Resilience | 0.07 | 0.059 | 0.103 | 1.185 | 0.238 | ±0.233 |
| 6 | (Constant) | -1.791 | 0.649 |  | -2.761** | 0.007 | ±2.566 |
|  | Sleep quality | 0.336 | 0.033 | 0.899 | 10.209** | <.001 | ±0.130 |
|  | General health | 0.097 | 0.071 | 0.086 | 1.359 | 0.177 | ±0.281 |
|  | Job tenure | -0.072 | 0.05 | -0.085 | -1.43 | 0.155 | ±0.200 |
|  | Stress | -0.356 | 0.056 | -0.4 | -6.417** | <.001 | ±0.219 |
|  | Resilience | -0.315 | 0.058 | -0.468 | -5.442** | <.001 | ±0.229 |

*p<0.001; *p<0.05; SE – standard error (of B); CI – confidence interval; each predictor produced a variance inflation factor <3 and a tolerance value ≥0.6; ---No applicable
